# Supplementary material for: An artificial spiking afferent nerve based on Mott memristors for neurorobotics
Source: Nat Commun. 2020 Jan 2;11:51. doi: 10.1038/s41467-019-13827-6 (PMC6940364; doi:10.1038/s41467-019-13827-6)
Supplement: Supplementary file 1 — Supplementary Information [file 41467_2019_13827_MOESM1_ESM.pdf]

Supplementary Information

An artificial spiking afferent nerve based on Mott memristors for neurorobotics

Zhang et al.

Supplementary Figures 1 − 12.

Supplementary Figures

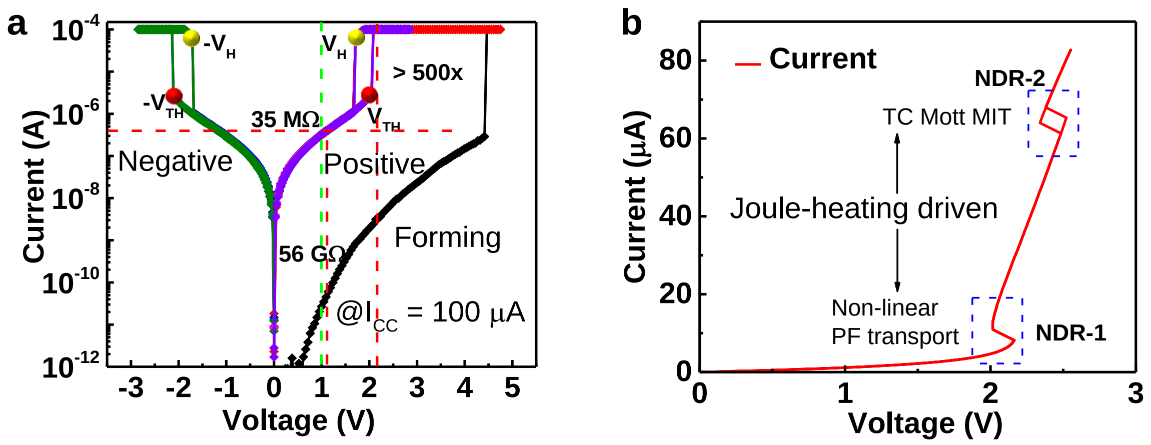

Supplementary Figure 1. I-V curves of the device under different sweep modes. a, The DC operation of the NbO_x device under voltage sweep. Before operation a DC sweep from 0 V to 5 V was required to irreversibly modify the low-bias resistance of the devices from the 56 GΩ (@1 V) virgin state to the 35 MΩ (@1 V) operational regime. After forming, the volatile switching behavior can be obtained in both positive and negative voltage with nearly identical absolute value of switching voltage. **b**, The DC operation of the NbO_x device under current sweep. Two negative differential resistance (NDR-1 & NDR-2) were observed. The NDR-1 is induced by the heating driven non-linear Poole-Frenkel (PF) transport driven instability and NDR-2 is caused by the temperature-controlled Mott metal-insulator-transition (TC Mott MIT). Both types of NDR are Joule-heating driven[1, 2].

Note: The single pinched hysteresis loop under voltage mode encompassed both NDR-1 and NDR-2[1]. So, in this work, a Joule-heating driven NDR mechanism is used to explain the switching behavior of the NbO_x device.

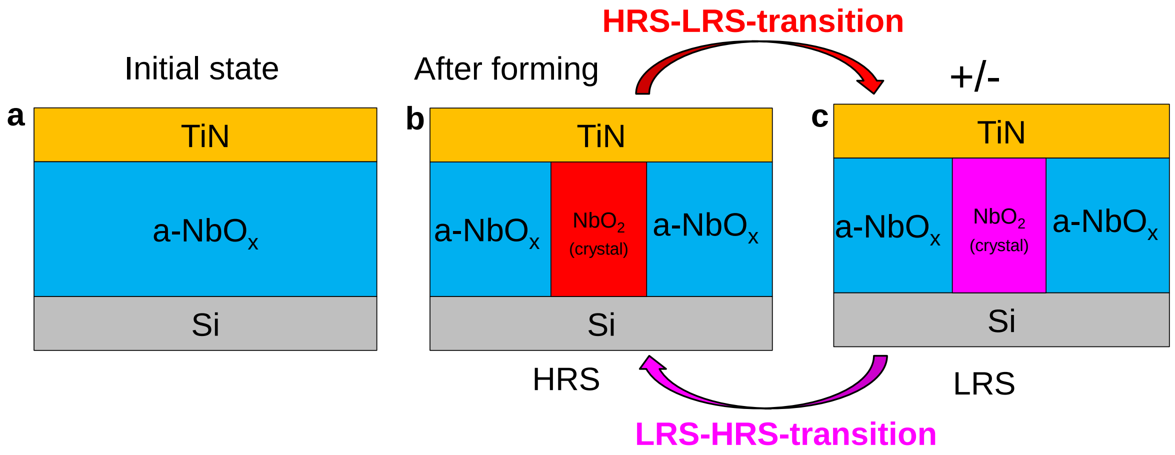

Supplementary Figure 2. The schematic of the switching mechanisms. a, The initial state of the NbOx device. **b**, **c**, After forming, a crystalline NbO_2 channel will be generated[1,3]. When the applied positive or negative voltage on TiN electrode surpasses a threshold switching voltage (V_{TH}) (Supplementary Figure 1 and Fig. 2k in main text), the NbO_2 channel switches to a low resistance state (LRS), then the channel switched back to its high resistance state (HRS) when the applied voltage below a hold voltage value (V_H) (Supplementary Figure 1 and Fig. 2k in main text). The detailed explanation of the physical mechanism is described in Supplementary Figure 1.

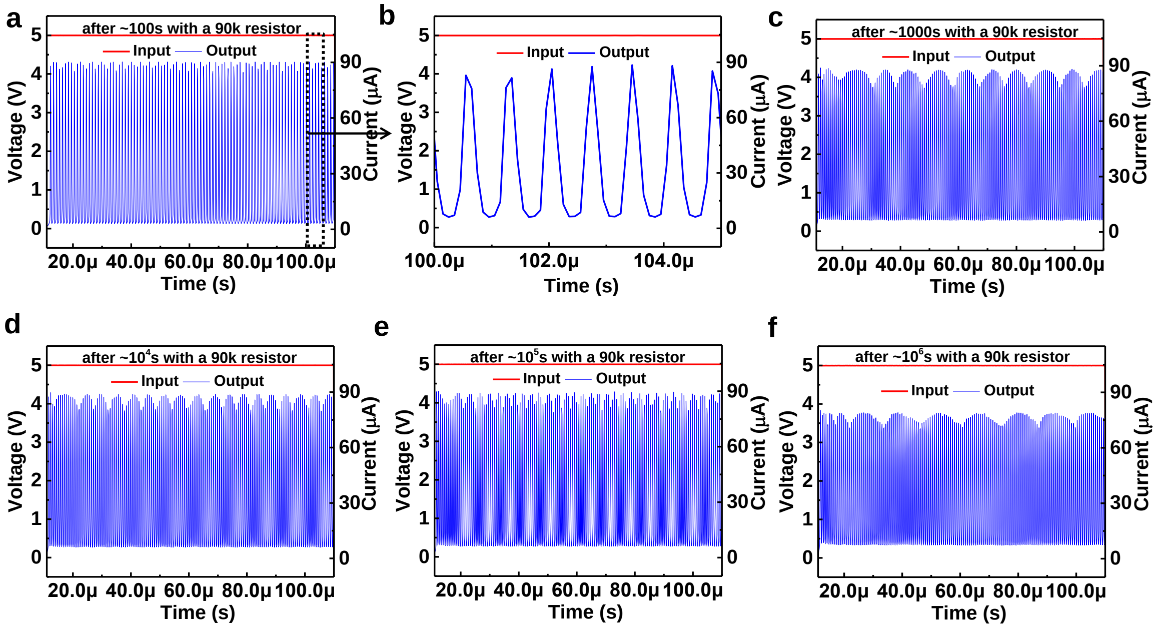

Supplementary Figure 3. Endurance measurement of the NbO_x Mott device. a-f, Tested endurance by successfully running the ASAN after **a** 100s, **c** 1000s, **d** 10^4 s, **e** 10^5 s, **f** 10^6 s at a period of < 1 μs, yields an endurance value $>10^{12}$; **b**, a closer view of **a**; during the endurance testing, a 90 k resistor was used. The results demonstrated that the NbO_x device could stand more than 10^{12} switching cycles and thus could support > 10^{12} times fire events.

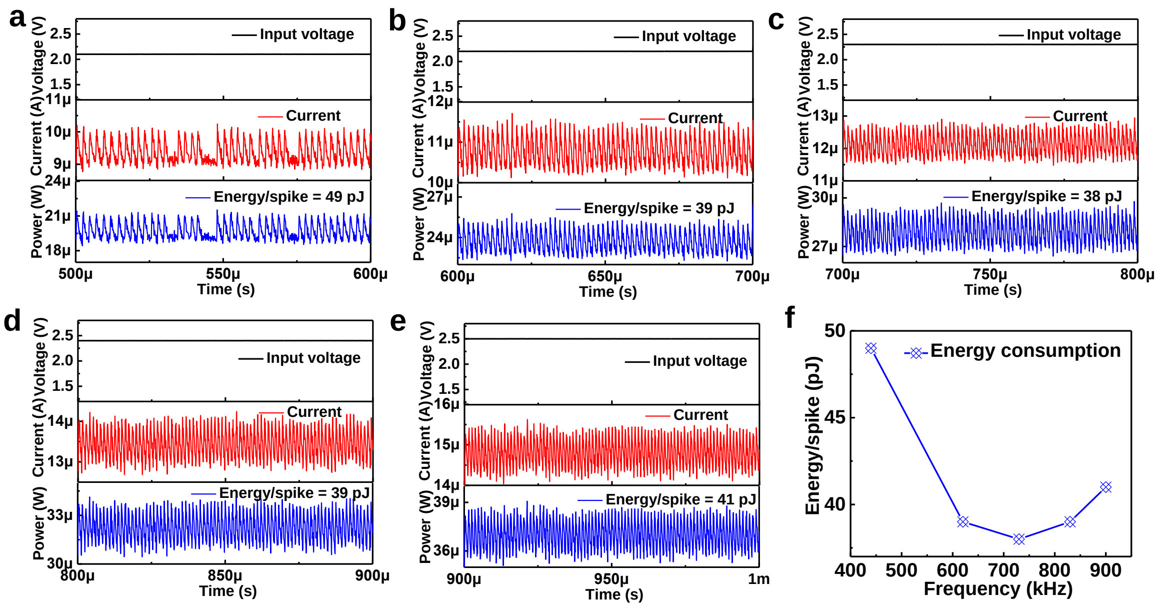

Supplementary Figure 4. The energy consumption of the afferent nerve. a-e, The energy consumption is extracted from **Fig. 3c**. The transient power is calculated by the multiply of input voltage and output current and the energy consumption for each spike was calculated by dividing the total consumption by the spike numbers. **f**, The energy consumption per spike under different spiking frequencies. The minimal energy consumption as low as ~ 38 pJ per spike event was achieved. And the energy consumption could be further reduced by using the NbO_x device with a lower threshold voltage and a testing circuit with smaller parasitic capacitance[2,3].

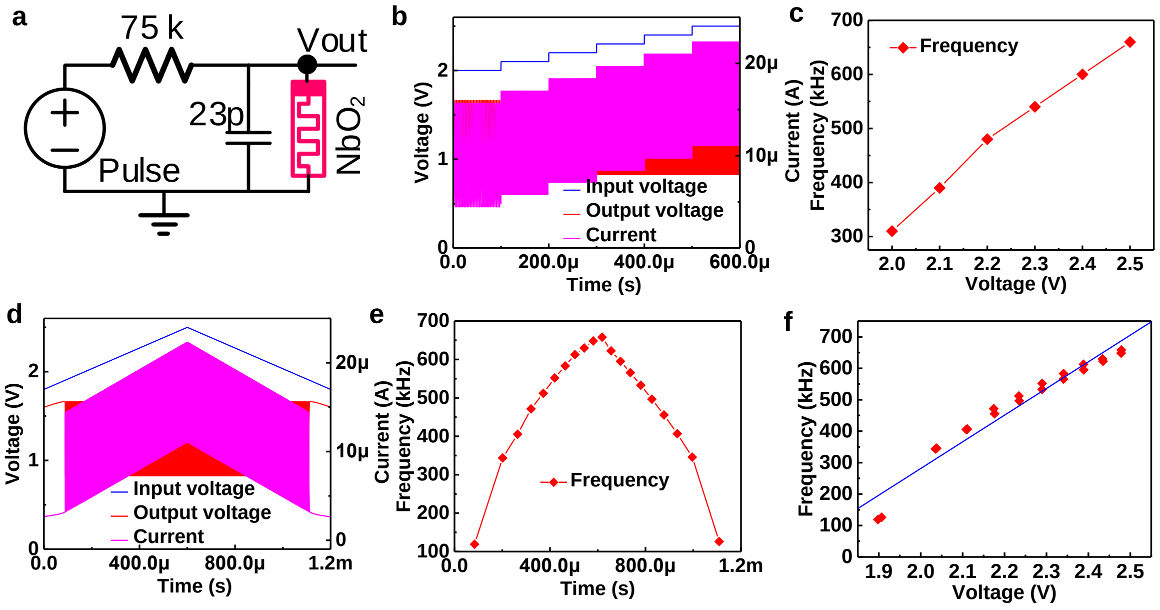

Supplementary Figure 5. Simulation results of the afferent nerve. a, The schematic of the simulation circuits, a 23-pF capacitor was used as the parallel parasitic capacitance. **b**, The simulation results under different voltage and total current flowing through the resistor. **c**, Related frequency-voltage curve. **d**, The simulated results under triangular pulse. **e**, & **f**, The extracted frequency response of the ASAN. The spiking frequency increases linearly with increasing the input voltages.

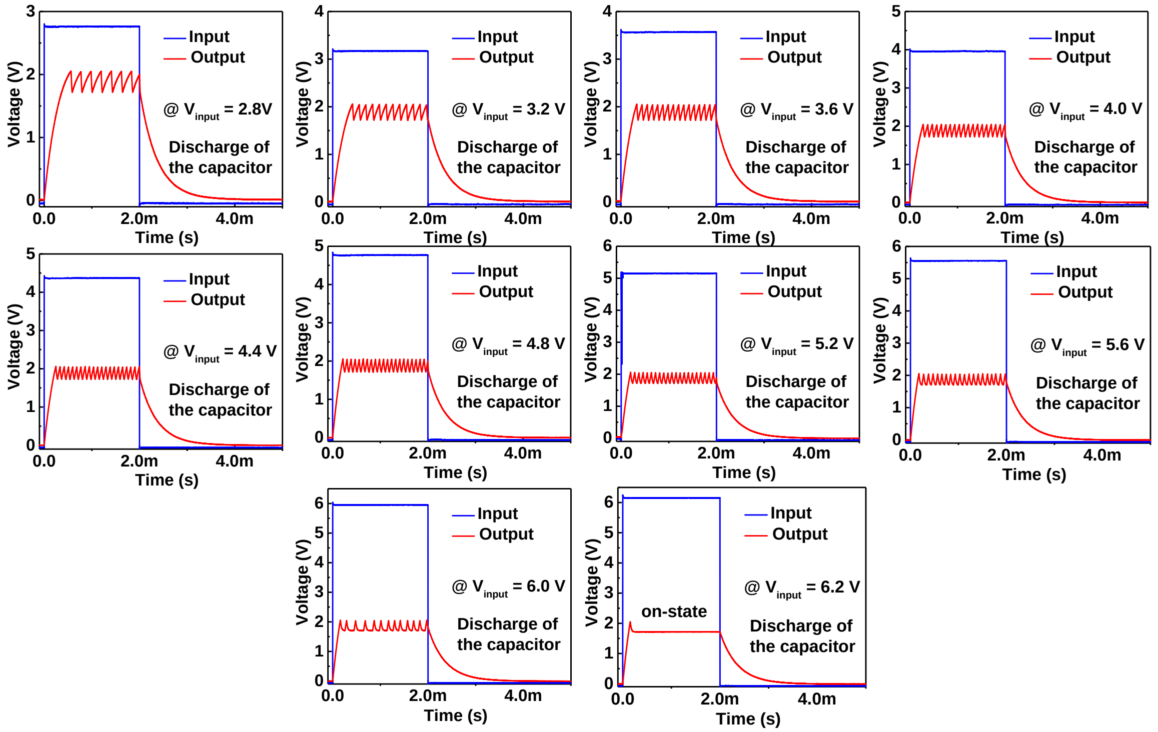

Supplementary Figure 6. The output results of the afferent nerve. This figure shows the additional data of the afferent nerve under different input voltages that not presented in **Fig. 4b**. When the input voltage increases to 6.2 V the NbO_x device will be stuck on, no oscillation behavior can be observed.

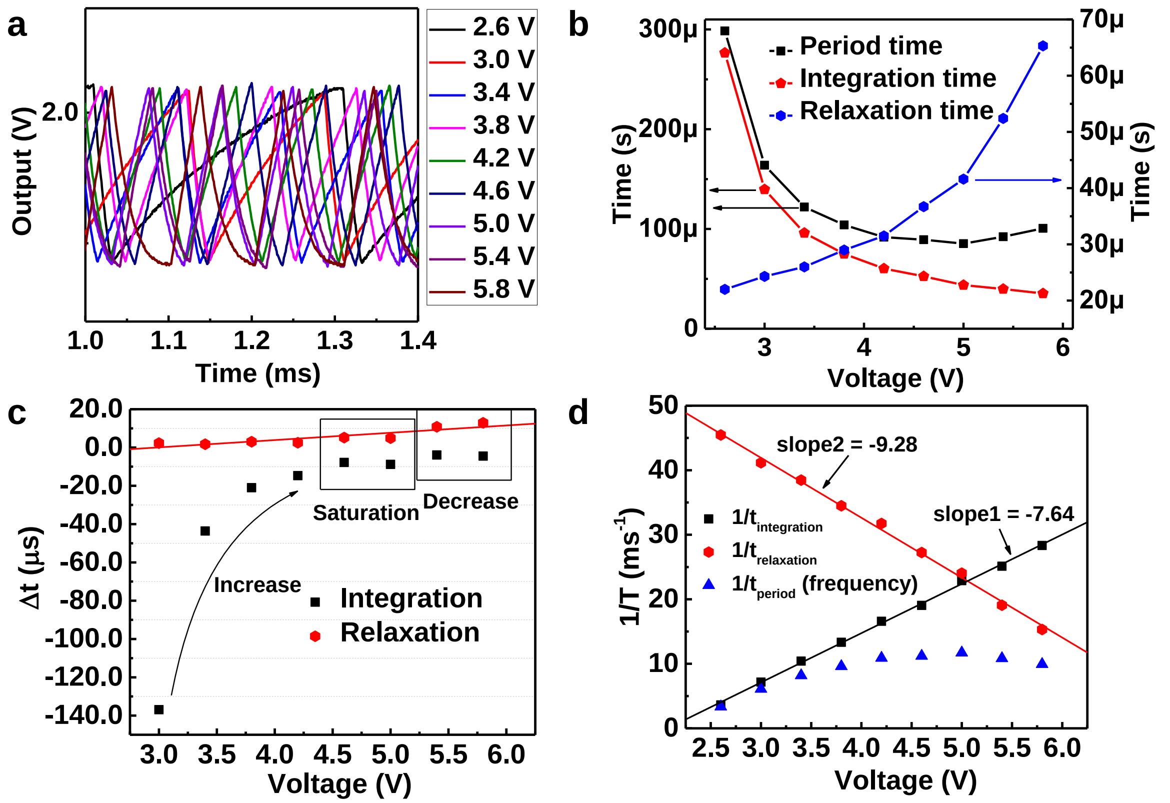

Supplementary Figure 7. Analysis of the integration time and relaxation time. a, A closer view of the integration and relaxation process of the artificial spiking afferent nerve (ASAN) under different input voltage. **b**, Extracted integration and relaxation time from **a**. The integration time decreases with increasing the input voltage, while the relaxation time increases. The whole period decreases firstly and then increases. **c**, The variation value of the integration and relaxation time under different input voltage. Each data point in **b** is compared to the previous adjacent data point. The abrupt decreased variation value of integration time and gradually increased relaxation variation value are observed. There are three stages, under lower input intensity, the integration variation value decreased abruptly. In this stage, the frequency is dominated by the integration time. Then the integration time variation value decreases to the saturation value, but the variation value of relaxation time continue increasing. When continue increase the input intensity, the

relaxation time dominates the frequency, which results in the decrease of the spiking frequency. **d**, The reciprocal value of the integration and relaxation time in **b** and the corresponding frequency. Both values have a linear relationship with input intensity. However, the relaxation time has a bigger slope absolute value than that of integration time, which indicates the finally dominant role of the relaxation time.

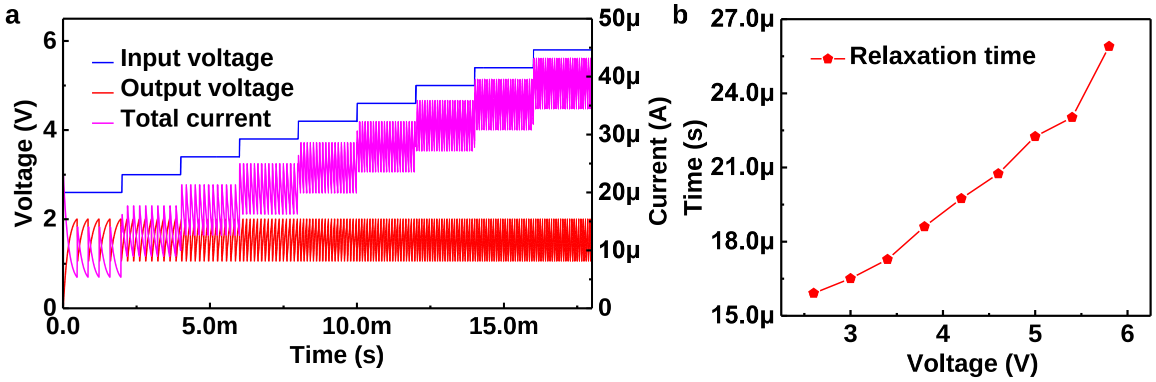

Supplementary Figure 8. Total current and relaxation time of the afferent nerve. a, The simulation results of the artificial spiking afferent nerve with a 4.7-nF capacitor. The total current in the circuit is demonstrated which increases with increasing the input voltages. **b**, The increased current under high voltage makes the relaxation time of the artificial spiking afferent nerve (ASAN) longer. The increasing current flow through the memristor leads to more Joule-heat in the memristor during the relaxation process, which makes the device dwell on its on-state for a longer time.

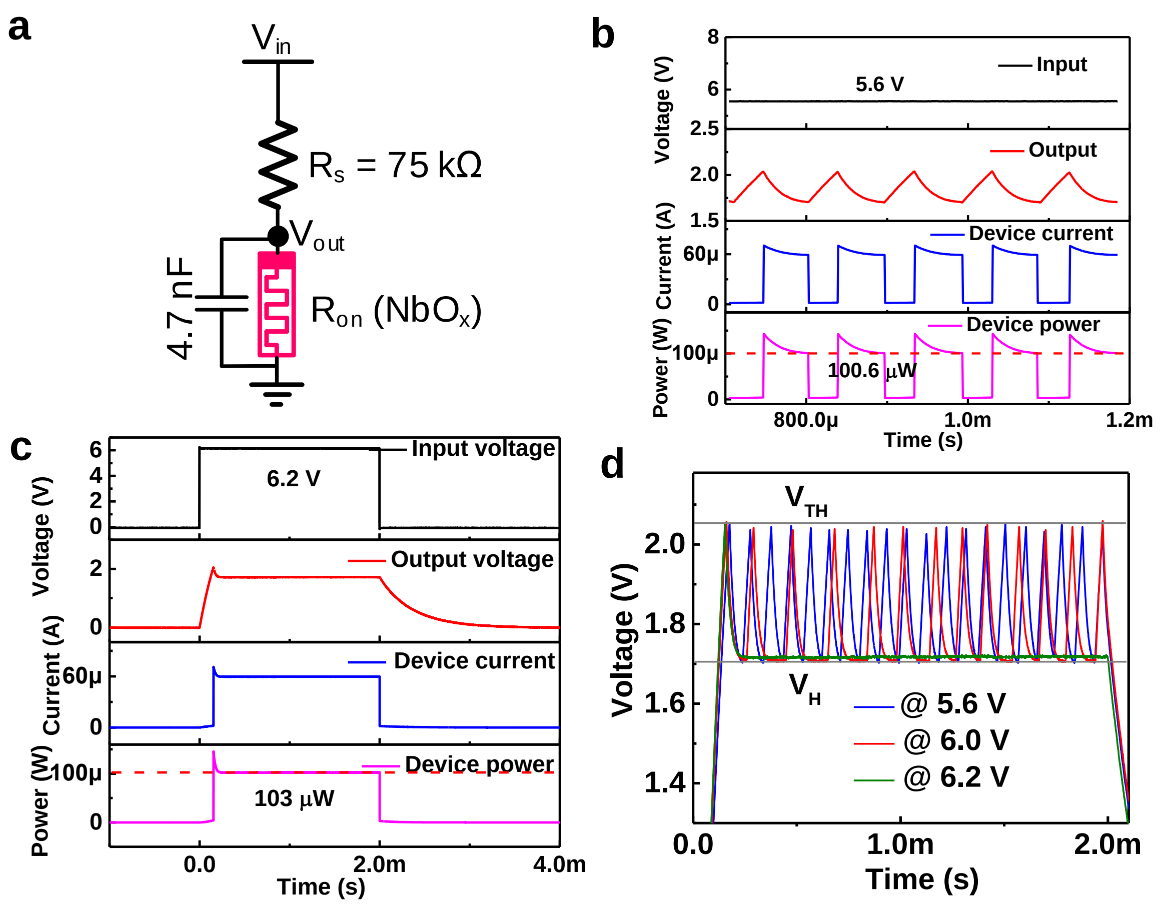

Supplementary Figure 9. Power consumption of the NbO_x device in afferent nerve. a, The schematic of the artificial spiking afferent nerve (ASAN) with a 4.7 nF capacitor. In the case of 6.2 V input voltage, according to Kirchoff's voltage law, $R_{on} = V_{out} * R_c / (V_{in} – V_{out})$ = 1.72 V * 75 kΩ / (6.2 V - 1.72 V) = 28.8 kΩ. The HRS value is estimated to be about 1 MΩ. **b**, The calculated transient current flowing through the NbO_x device and the power consumption on the device under a 5.6 V input voltage of the ASAN. 100.6 μW hold power value is observed, which should be smaller in real due to the nonlinear I-V characteristics of the NbO_x device. **c**, The calculated transient current flowing through the NbO_x device and the power consumption on the device under a 6.2 V input voltage of the ASAN. About 106 μW hold power is observed, which is enough to hold the device in its "on-state". **d**, Comparison of the oscillation behavior under 5.6 V, 6.0 V and 6.2 V input voltages, respectively. The output voltage of the ASAN under 6.2 V input voltage is

always larger than the V_H of the NbO_x device, which results in the device dwells in its "on-state".

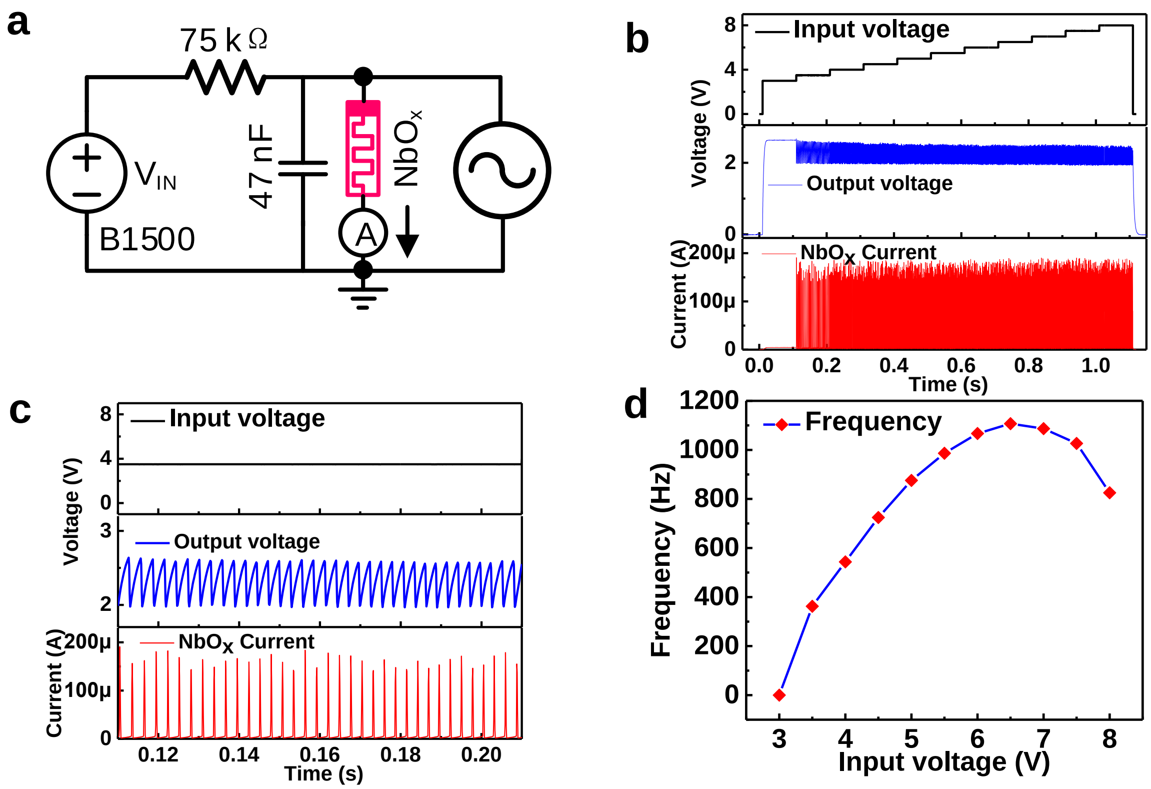

Supplementary Figure 10. Afferent nerve spiking behavior with a 47 nF capacitor. a, Schematic of the test circuits. **b**, The tested data of the input voltages, output spikes, and corresponding current signal flowing through the NbO_x device. **c**, Zoom-in views of **b**. **d**, The spiking frequency as a function of input voltages. A spiking range from 0 to 1100 Hz matching with the human nervous system (1 ~ 1000 Hz) is obtained.

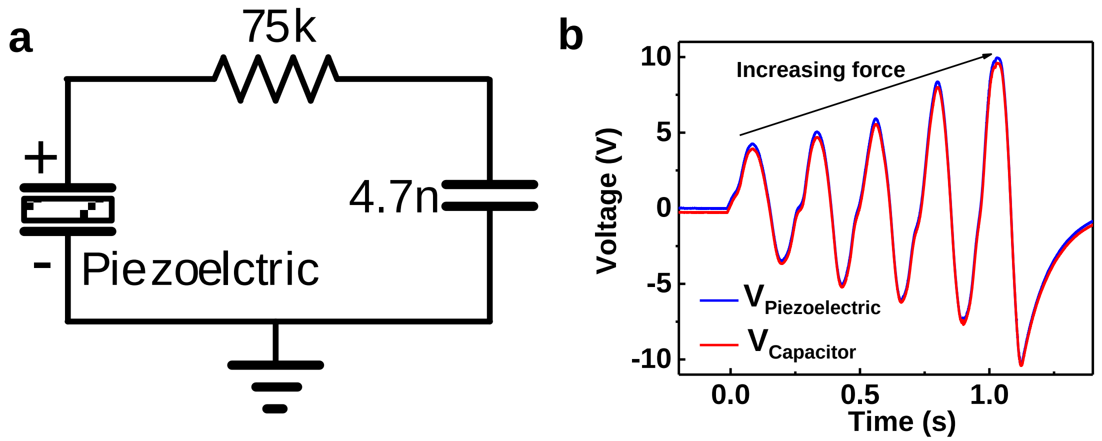

Supplementary Figure 11. The characteristics of the piezoelectric device. a, A piezoelectric device is in series with a resistor and a capacitor. **b**, Five continuous forces with incremental intensity were applied on piezoelectric device[4]. The voltages on piezoelectric device and the capacitor were measured.

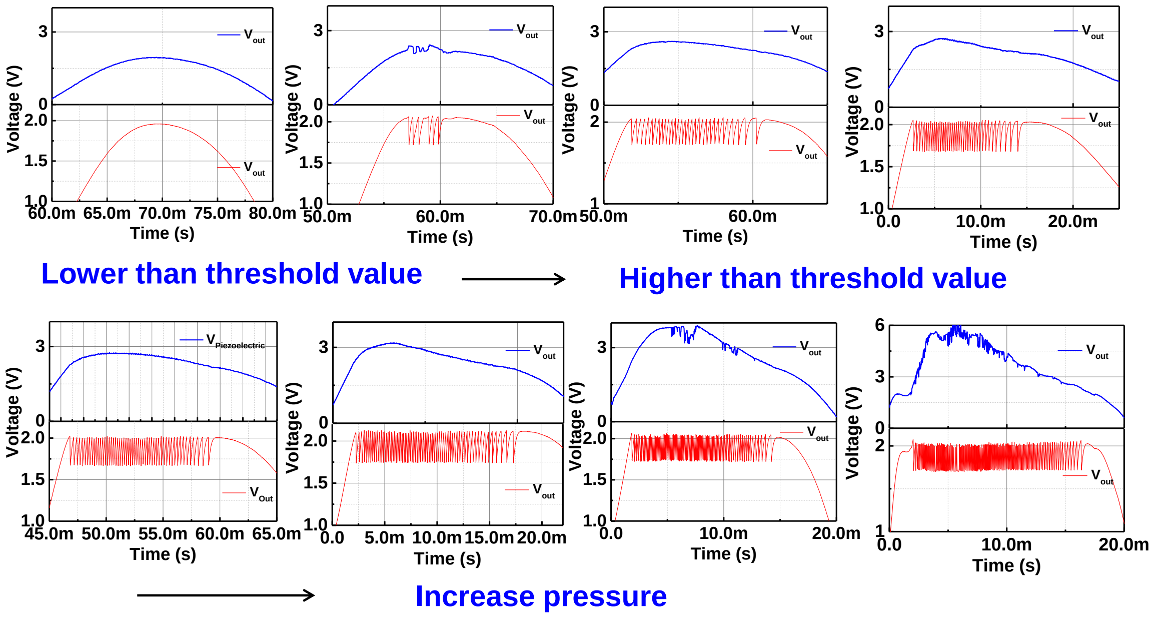

Supplementary Figure 12. **More experimental data of the mechanoreceptor system.** Various output frequency under different forces of the artificial spiking mechanoreceptor system, the oscillation frequency becomes dense with increasing the force pressure.

Supplementary References

1. Kumar, S. et al. Physical origins of current and temperature controlled negative differential resistances in NbO_2, *Nat Commun*. **8**, 658 (2017).
2. Kumar, S., Strachan, J.P. & Williams RS. Chaotic dynamics in nanoscale NbO_2 Mott memristors for analogue computing, *Nature* **548**, 318-321 (2017).
3. Pickett, M.D. & Williams, R.S. Sub-100 fJ and sub-nanosecond thermally driven threshold switching in niobium oxide crosspoint nanodevices, *Nanotechnol.* **23**, 215202 (2012).
4. Zhang, G. et al. Novel Piezoelectric Paper-Based Flexible Nanogenerators Composed of $BaTiO_3$ Nanoparticles and Bacterial Cellulose, *Adv. Sci.* **3**, 1500257 (2016).
